# The Development of Motor Self-Regulation in Ravens

**DOI:** 10.3389/fpsyg.2017.02100

**Published:** 2017-11-29

**Authors:** Can Kabadayi, Ivo Jacobs, Mathias Osvath

**Affiliations:** Department of Cognitive Science, Lund University, Lund, Sweden

**Keywords:** inhibitory control, motor self-regulation, comparative developmental studies, corvid cognition, detour behavior, cylinder task

## Abstract

Inhibitory control refers to the ability to stop impulses in favor of more appropriate behavior, and it constitutes one of the underlying cognitive functions associated with cognitive flexibility. Much attention has been given to cross-species comparisons of inhibitory control; however, less is known about how and when these abilities develop. Mapping the ontogeny of inhibitory control in different species may therefore reveal foundational elements behind cognitive processes and their evolution. In this study, we tested the development of motor self-regulation in raven chicks (*Corvus corax*), using two detour tasks that required inhibition of motor impulses to directly reach for a visible reward behind a barrier. One task included a mesh barrier, which partly occluded the reward, and the other task used a completely transparent barrier, the cylinder task. The results suggest that the more visible a reward is, the more difficult it is to inhibit motor impulses toward it, and further, that this inhibitory challenge gradually decreases during development. The mesh barrier is reliably detoured before the animals pass the task with the wholly transparent cylinder. As the majority of the birds begun testing as nestlings, and as we provided them with experiences they normally would not receive in a nest, it is likely that they showed the earliest possible onset of these skills. A control subject, tested at a later age, showed that the mesh detours required no particular training, but that tasks including complete transparency likely require more specific experiences. Adult ravens without explicit training are highly proficient in inhibitory detour tasks, and, together with chimpanzees, they are the best performers of all tested species in the cylinder task. Our results suggest that their skills develop early in life, around their third month. Their developmental pattern of inhibitory skills for detours resembles that of children and rhesus macaques, albeit the pace of development is markedly faster in ravens. Investigating the development of cognition is crucial to understanding its foundations within and across species.

## Introduction

Inhibitory control is a core component of executive functions, and can be defined as inhibiting prepotent responses in favor of more appropriate and productive actions for a given situation (Casey et al., 2011; Diamond, 2013). Motor self-regulation, which requires stopping a prepotent but counter-productive movement (Beran, 2015), is an essential aspect of inhibitory control. It is thought to underpin more taxing inhibitory processes, as they cannot be expressed if such basic self-regulation is weak. Given its importance, comparative investigations of motor self-regulation generate insights into aspects of cognitive evolution, such as whether this skill correlates with more complex cognition and ecological and phylogenetic factors (MacLean et al., 2014; Meier et al., 2017).

Recent years have seen several comparative investigations of motor self-regulation on a wide range of species, using detour tasks around transparent barriers (Amici et al., 2008; Vlamings et al., 2010; MacLean et al., 2014; Kabadayi et al., 2016). In order to reach for the visible reward behind the barrier, the subject must inhibit the prepotent motor response of directly reaching for the reward, and instead detour around the barrier (Diamond, 1990). While such comparisons may reflect different degrees of motor self-regulation abilities across taxa, the comparisons are often based on the average scores of adult individuals over few trials, and do not inform how the skill develops in different lineages.

Extending the comparisons to the developmental patterns may reveal important clues to cognitive evolution. Species differences in mature characters often result from alterations in developmental pathways (Gould, 2002; West-Eberhard, 2003; Rosati et al., 2014); thus, comparing the pace and pattern of cognitive development between species may explain variation in mature cognition. Although both comparative and developmental studies have long pedigrees, they are rarely integrated, and important insights may be overlooked (Gomez, 2005; Rosati et al., 2014). For example, inhibitory control develops more slowly in bonobos than chimpanzees, which may explain why adult chimpanzees display higher levels of self-control compared to bonobos (Rosati et al., 2007, 2014; Herrmann et al., 2010; Wobber et al., 2010).

Motor self-regulation tasks requiring detours around barriers have rarely been used in developmental studies. Human infants show a clear developmental progression between 6 and 12 months in reaching around transparent barriers (Diamond and Gilbert, 1989). Initially they find it difficult to reach through the side opening of a transparent box and instead attempt to directly reach for the visible but blocked reward. They gradually overcome this difficulty and execute detours around transparent barriers by the end of their first year (Diamond, 1990). Rhesus macaques (*Macaca mulatta*) go through a similar developmental trajectory between 1 and 4 months (Diamond and Goldman-Rakic, 1986). At a certain point in development, once they have attained object permanence (Piaget, 1954), both humans and rhesus macaques are proficient in detouring opaque barriers while they experience problems in detours around identical but transparent barriers, likely because the visible reward creates a strong motivational pull for a direct reach (Lockman, 1984; Diamond, 1991). Thus, the improvement in detour performance around transparent barriers is attributable to the development of motor self-regulation skills (Diamond, 1990).

There are currently no developmental studies on motor self-regulation involving detours in birds. Ravens (*Corvus corax*) are good candidates for such developmental investigation because they excel at inhibition tasks as adults, and are paralleled only by chimpanzees in a motor self-regulation task involving detours around transparent barriers (Kabadayi et al., 2016). Ravens are also renowned for their cognitive skills in various domains (Güntürkün and Bugnyar, 2016), including self-control (Dufour et al., 2012), and complex planning (Kabadayi and Osvath, 2017), often matching great apes in their abilities. Thus, investigating the development of motor self-regulation in ravens might reveal developmental patterns similar to those in other cognitively proficient mammals (Diamond, 1990; Rosati et al., 2014). This in turn can have implications for the understanding of independent evolution of complex cognition, i.e., whether different lineages – such as corvids and primates – undergo similar developmental stages when building complex cognition (Osvath et al., 2014).

**FIGURE 1:**
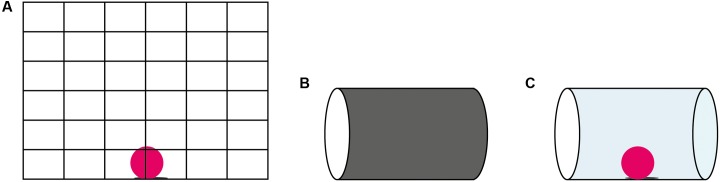
The materials for the detour tasks: **(A)** the mesh barrier; **(B)** the opaque cylinder; **(C)** the transparent cylinder.

We tested the development of motor self-regulation in raven chicks using two detour tasks: the transparent cylinder task and the mesh barrier task (**Figure 1**). The transparent cylinder task has recently become a benchmark test for motor self-regulation and has been administered to a wide range of species, including adult ravens (MacLean et al., 2014; Kabadayi et al., 2016). It is functionally identical to the object-retrieval task, which has been used in previous developmental studies in human and rhesus macaque infants (Diamond, 1990). The mesh barrier task, which has a long history within comparative psychology, was modeled after previous studies that used grid or wire barriers to study detour behaviors (Köhler, 1927).

In the transparent cylinder task, a reward is placed at the center of a transparent cylinder with two openings at its ends. The subject must inhibit the response of directly reaching for the visible but blocked reward and instead detour through either of the two openings in the ends of the cylinder. An opaque cylinder is used as a control. Opaque controls have also been used in functionally similar tasks in previous developmental studies on human and rhesus macaque infants (Diamond, 1990). Because the opaque and transparent cylinder differ only in reward visibility, and require identical detours to obtain the reward, a lower performance on the transparent task compared to the opaque task reflects the difficulty to motor self-regulate. It is then likely that the visibility of the reward creates a prepotent tendency to directly reach for it.

The mesh barrier task, used in this study, consists of a grid barrier that required the animal to walk around the barrier in order to obtain the reward. Since the mesh barrier partly occludes the reward by its wires, it is arguably less taxing than the transparent cylinder where the whole reward is clearly visible, and many species execute more efficient detours around mesh barriers than transparent ones (Regolin et al., 1994; Lockman and Adams, 2001; Vallortigara and Regolin, 2002; Juszczak and Miller, 2016). The cylinder task, with its transparent surface, is likely also more artificial than the mesh barrier task, in the sense that complete transparency is an unusual property of the environment. Whereas the animal might early on learn that it cannot walk through opaque objects even if it sees the goal, it might require specific experiences with transparent surfaces to understand the affordances of such materials.

Therefore, we predicted the ravens’ motor self-regulatory development would be reflected in their performances on these tasks: they will perform better and succeed earlier on the mesh barrier task compared to the transparent cylinder task, and they will perform most accurately on the opaque cylinder task as the invisible reward behind the opaque barrier creates no motor self-regulation challenges. Detouring around the opaque barrier instead requires object permanence as it involves orienting toward an object out of sight. Ravens become successful in searching and retrieving fully hidden objects (object permanence) around 8 weeks of age (Bugnyar et al., 2007). Thus, we predicted they would succeed in detouring around the opaque barrier around this time. However, we started the tests as soon as the ravens fledged (on their 6th week), in order to capture a wider possible period of motor self-regulation development.

## Materials and Methods

### Subjects

Five raven chicks (four females) took part in this study. One of these five chicks (a female) acted as a control for the role of experience in the development of detour behavior. The testing of this chick began at the same age as when the other four individuals had passed the detour tasks. Four ravens were hand-raised at the Lund University Corvid Cognition Station after being removed from four different nests in the wild. At the time we obtained them two females were 5 weeks old, one female was 4 weeks old and the male was 2 weeks old. We used the time of fledging when estimating the age of the chicks. Ravens fledge around 44 days after hatching with a few days of variation (based on the information from five different nests from the previous 4 years as well as the fledging date of the hand-raised ravens at the station). This estimation was accompanied and independently corroborated by the before-fledging age estimations of the caretakers who had years of experience in hand-raising ravens and had photographic collections of ravens of different ages. After the birds had fledged their artificial nests were kept inside a large aviary with a group of five adult ravens. With the exception of the control bird (which began testing at week 10), testing began during their 6th week post-hatching and finished when they were around 11 weeks old. When the testing began, the ravens would always return to their nests after bouts of movements and short flights, i.e., this was in the earliest stage of fledging. The female chick that acted as control was hatched and hand-raised in captivity at a zoo (Skånes Djurpark, Sweden). She was removed from the nest at week 3 (post-hatching) and hand raised by caretakers. She had no experience with transparent objects prior to testing, which was conducted individually in a zoo aviary with metal bars as outer walls that could not be detoured. The other birds had no experience of anything like the materials used in the experiments, until we provided them. Before fledging they lived in an artificial nest, with nest materials from twigs and hay, and they were lifted from the nest to a table with the testing materials.

### Materials and Procedures

A transparent cylinder, an opaque cylinder and a mesh barrier were used (**Figure 1**). The size of the cylinders followed the criterion set by MacLean et al. (2014): sufficiently long (12 cm) so that the birds can obtain the reward at the center of the cylinder by inserting their heads through the opening, but not too large (diameter of 9 cm) so that the birds cannot enter the cylinder with their whole body. The mesh barrier (51 cm × 46 cm) with evenly spaced horizontal and vertical grids (3 cm × 5 cm) required the animal to move its entire body around the barrier to reach the reward (**Figure 1**). All apparatuses were attached to a wooden support and placed on the testing table. The testing table was placed in the same room as the artificial nest and was hence familiar to the subjects. However, during testing the nest was placed so the non-tested individuals could not observe the experiment. The chicks were tested weekly on all tasks, and their progression was tracked until they performed at 100% correct for two consecutive weeks. All birds received 10 trials in each of the three tasks every week in randomized order. Testing took place on maximum 2 days a week.

In both the transparent and opaque cylinder task, the experimenter placed a desirable reward (a colorful toy or a food item) in the middle of the cylinder while the subject was observing, and thereafter the subject could approach the apparatus to retrieve it. The toy was replaced by food once the birds could feed on their own. A correct response for the transparent cylinder was coded when the reward was retrieved through either of the side openings without any prior contact with the long side surface of the cylinder. An incorrect response was coded when contact was made with the long side of the cylinder (likely in an attempt to directly reach for the reward).

In the opaque cylinder a correct response was coded as retrieving the reward from either of the openings without any prior contact with the long side, while an incorrect response was coded either if contact was made with the long side, or if no attempt of retrieving the reward had been made within 10 s after baiting. This 10-s criterion was added to the opaque cylinder task because ravens lack object permanence at certain ages, and therefore would not retrieve the reward – precluding data collection at this age. It also allows for comparing the development of object permanence to that of motor self-regulation. Note that the use of the cylinders differed slightly from other studies including these tasks (e.g., MacLean et al., 2014; Kabadayi et al., 2016). In other studies, the subject is only allowed to proceed to the transparent cylinder if it has reliably passed the opaque one (usually in four out of five trials). However, in this study the subjects were tested on the transparent cylinder regardless of whether they had passed the opaque one or not. This was done for two reasons: (1) the subjects might be able to pass the transparent cylinder before they have developed object permanence; but (2) if they do not, it is of interest to know whether they pass the opaque cylinder earlier in development than the transparent one, as this would indicate that the visual reward creates inhibitory challenges.

In the mesh barrier task, a reward was placed on the other side of the barrier while the subject was observing. The reward was placed far enough (11 cm) from the barrier to prevent the bird from reaching it through the mesh. A correct response was coded when the bird moved around the barrier and reached the reward. An incorrect response was coded as an attempt to directly reach for the reward through the mesh.

Regardless of their initial response, the subjects were allowed to retrieve the reward in every task. A generalized linear mixed-effect regression model was constructed to investigate the effect of the task, age (in weeks) and their interaction on the responses, which was a binary outcome variable (correct or incorrect). In order to account for the repeated measurements of the same individual, individual birds were added to the model as random effects. Statistical analyses were conducted in R, version 3.1.3 (R Core Team, 2015).

For the raven chick who was tested as a control, testing began at the earliest week where all four ravens succeeded on all tasks. The procedure and coding criterion were identical, except that the reward always was a food item.

The protocol and design was approved by the regional ethics board for animal research in the county of Skåne (No. M 333-12). For the chick that acted as control, which was tested in the Skåne zoo (Skånes Djurpark), no approval was needed according to the Swedish regulations. For all birds, we followed the guidelines for the treatment of animals in behavioral research, by the Association for the Study of Animal Behaviour (ASAB).

## Results

**FIGURE 2:**
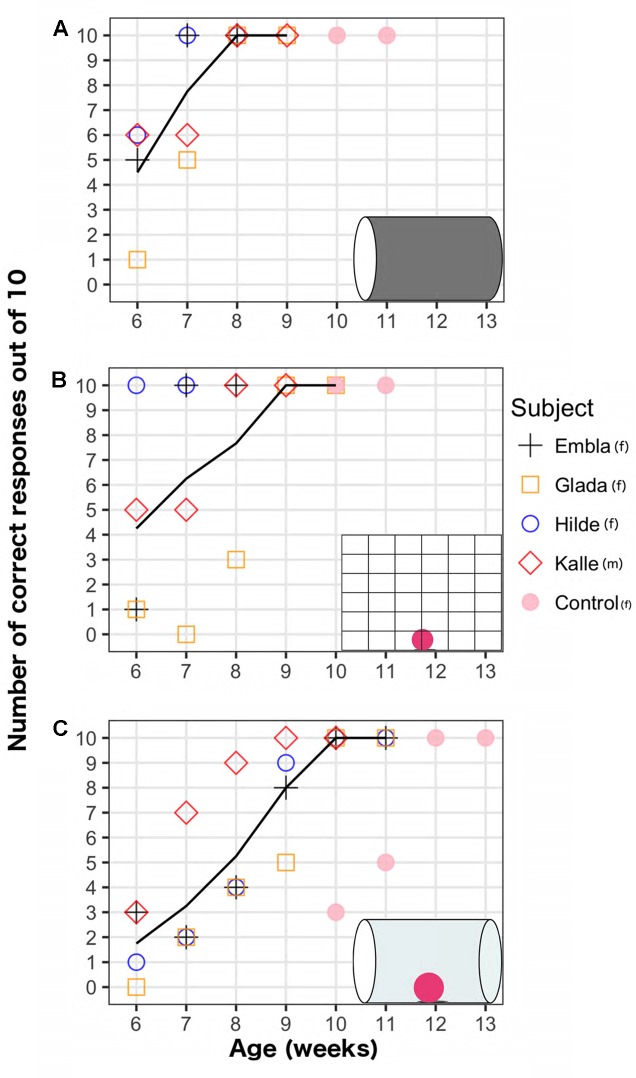
Weekly performance of five raven chicks on three detour tasks (shown in insets): **(A)** Opaque cylinder task **(B)** Mesh barrier task **(C)** Transparent cylinder task. Lines show average scores across weeks for four subjects that began testing on week 6, excluding the control bird that began testing on week 10.

On average, the four ravens first reached 100% performance in the opaque cylinder task in week 7.5 (minimum: 7, maximum: 8); the mesh barrier task in week 7.5 (minimum: 6, maximum: 9); and the transparent cylinder task in week 9.75 (minimum: 9, maximum: 10). The weekly development of the performance on the two motor self-regulation tasks and the opaque cylinder task is shown in **Figure 2**. Regression analyses revealed that the motor self-regulatory responses in both tasks increased significantly over weeks (transparent cylinder task: EST = 1.36, *SE* = 0.21, *z* = 6.65, *p* < 0.001, mesh barrier task: EST = 1.35, *SE* = 0.28, *z* = 4.76, *p* < 0.001). The performance levels in the opaque cylinder also improved significantly over weeks (EST = 2.42, *SE* = 0.48, *z* = 5.00, *p* < 0.001). In the first week of testing, performance levels in the transparent cylinder were significantly worse than the opaque cylinder (EST = -1.81, *SE* = 0.49, *z* = -3.70, *p* < 0.001) and the mesh barrier (EST = -1.66, *SE* = 0.47, *z* = -3.54, *p* < 0.001).

The performances in the transparent cylinder increased less steeply over time compared to the opaque cylinder (EST = -1.054, *SE* = 0.48, *z* = -2.17, *p* = 0.029), but there was no significant difference between the performance slopes on the transparent cylinder and mesh barrier (EST = -0.012, *SE* = 0.29, *z* = -0.043, *p* = 0.966). Performance levels in the opaque cylinder were higher than in the transparent cylinder in the same week (except for the male in week 7 and until the ravens reached ceiling performance in both tasks). There was greater individual variation in the mesh barrier as every individual reached the criterion on different weeks (weeks 6, 7, 8, and 9, respectively). The bird that succeeded earliest on the mesh barrier (week 6) did not succeed earlier than the other birds on the transparent cylinder. All birds succeeded on the mesh barrier before the transparent cylinder. One individual succeeded on the mesh barrier a week before the opaque cylinder. For all three tasks, once the subjects reached 100% performance on a given week, their performance on the same task always remained 100% in the following week, after which the testing ended. By week 10, all four birds succeeded in all tasks.

Because all four birds succeeded on all three detour tasks latest at 10 weeks, we started testing a naïve chick with all three tasks when she was 10 weeks old (Unfortunately, only one chick was available). Her performance on the mesh barrier and the opaque cylinder was 100% already on the first week of testing, and remained 100% on the subsequent week of testing. She reached 100% on the transparent cylinder in the third week of testing – when she was 12-weeks old – with a first week performance of 3 out of 10, and a second week performance of 5 out of 10. Her performance remained 100% on the subsequent week (week 13).

## Discussion

As predicted, greater reward visibility posed a bigger challenge to the ravens, as reflected in the order in which they succeeded on the tasks. At an earlier developmental stage, the ravens performed worse on the transparent barrier than the mesh barrier. This difference was probably due to the stronger perceptual pull by the more visible reward behind the transparent barrier. Similarly, the successes on the transparent cylinder were initially fewer than those on the opaque cylinder. When the ravens were successful in retrieving the reward from the opaque cylinder, they had difficulty executing the same behavior on the transparent cylinder for the next few weeks. Thus, the gradual increase in performance in the transparent cylinder task can be attributed to the development of motor self-regulation.

Similar developmental patterns using transparent and opaque barriers have been found in human infants between 6 and 12 months and rhesus macaque infants between 1 and 4 months (Lockman, 1984; Diamond, 1990; Lockman and Adams, 2001). During these months, both human and rhesus macaque infants have difficulties in making detours around a transparent barrier while they are proficient on opaque barriers. Thus, the developmental pattern of motor self-regulation in ravens resembles that of children and rhesus macaques, albeit the rate is considerably accelerated in absolute time. Our results also support previous findings of that reduced reward visibility improves detour performance (human infants: Lockman and Adams, 2001; Noland, 2008; chickens: Regolin et al., 1994; mice: Juszczak and Miller, 2016; dogs: Chapuis et al., 1983; cats: Poucet et al., 1983). Ravens succeeded in detouring around the opaque barrier around 8-week post-hatch, which coincides with the time they achieve object permanence skills (uncovering a fully hidden object; Bugnyar et al., 2007). This developmental pattern is also found in human infants, albeit at a later age (Lockman, 1984). However, there are possibly some task differences between species: ravens had to move their whole body to obtain the reward, but the primates could reach for the reward behind the barrier using their hands only while sitting in front of the barrier (Diamond, 1990).

Improvement on detour tasks across trials is a common finding in many species, even in adults; indeed, detour problems have been used to study various learning processes (Wyrwicka, 1959; Scholes and Wheaton, 1966; Boogert et al., 2011; Vernouillet et al., 2016). However, the results in this study likely do not reflect merely a task learning phenomenon decoupled from a developmental one. For example, the 10-week old raven that was tested for the first time immediately performed flawlessly on the mesh barrier task. That this bird did not need any specific experience with the materials or tests to pass suggests that the improvement on the mesh barrier task for the other four birds reflected largely a developmental phenomenon. This bird might have had general experience from moving about outside the nest and encountering situations where it could not reach goals due to physical obstacles. The other birds had little such experience when they were tested the first time.

When it comes to the transparent cylinder task, the 10-week old bird that was tested for the first time did not reach the criterion immediately but needed some experience until she succeeded at 12 weeks post-fledging. This might be due to the fact that transparent objects are highly artificial and they pose conflicting visual-tactile information. Accordingly, it takes more experience to understand them as a barrier (Bojczyk and Corbetta, 2004). And yet, this individual was 100% correct on the transparent cylinder task already in her third week of testing. Her performance could of course also reflect slight variations in the speed of cognitive development between individuals. Among the other four birds that started the tests earlier, three individuals required 5 weeks and one individual required 4 weeks of testing until they reached 100%. This could suggest that the performance on the transparent cylinder increases faster when tests start at a later stage, possibly due to an interaction between experience and development.

Adult ravens, including one sub-adult (a 1-year-old), performed 100% correct on the transparent cylinder task without training, indicating that they do not need any specific training on the materials for succeeding (Kabadayi et al., 2016). The finding that untrained adult ravens perform perfectly on the transparent cylinder task suggest they already have a fully developed motor self-regulation ability in relation to detours by the age of one. Thus, the improvement of the raven chicks on the transparent cylinder task points to developmental maturation. However, it is well-known that repeated task exposure plays an important role in development – including detours around transparent barriers (Bojczyk and Corbetta, 2004). It is likely that birds with more experience with transparency succeed earlier than those without (as hinted at by the control bird). Consistent with this idea, human infants that were tested repeatedly throughout development (longitudinal group) succeeded in taking detours around transparent barriers about 2–4 weeks earlier than infants in the cross-sectional group that did not receive repeated trials (Diamond, 1988). Cognitive development always occurs in relation to an environment, so it is difficult to pinpoint the exact timings of different skills on a species level. Nonetheless, it is possible to find the earliest emergence of a particular skill when comparing subjects with different experiences. It is possible that the four ravens tested longitudinally showed the earliest possible onset of these skills as they were specifically provided the tasks early on.

Despite difficulties with demarcating learning from development – as learning is part of ontogeny – there are further reasons to think that a learning account decoupled from other cognitive maturation offers a less convincing explanation. The order in which the detour tasks were solved, based on increased reward visibility, is predicted from a gradual development of inhibitory skills. The motor self-regulation account is also consistent with the finding that ravens found it harder to make detours around transparent barriers while being proficient in detouring identical but opaque ones. Such an “opaque advantage” during a certain developmental period suggests a knowing/acting mismatch, which is common in other types of inhibition problems. That is, the animal knows the task rule (detour around the barrier) but cannot act on that rule, because the visible reward creates a strong incentive for a direct reach. The development of motor self-regulatory skills thus frees this knowledge, and detours can be executed by inhibiting motor responses instigated by the visible reward.

In summary, we found that ravens follow a developmental trajectory in motor self-regulation similar to that of primates, albeit at a considerably faster pace in absolute time. This higher speed is consistent with previous developmental studies on other cognitive skills in ravens, such as object permanence (Bugnyar et al., 2007) and gaze following (Schloegl et al., 2007). Given the vast phylogenetic separation between birds and mammals, the results may shed glimpses of light on the questions surrounding independent evolution of complex cognition. If cognitive skills are similar in adult ravens and apes, and if these skills appear to rely on similar developmental pathways, it might suggest that parallelism, instead of convergence, is the evolutionary mechanism (Osvath et al., 2014), however, it could also suggest that there is only one way to developmentally produce certain forms of cognition. As both task affordances and contextual factors might affect performance, future developmental studies should also include other motor self-regulation tasks – such as the stop-signal task – to gain a more detailed understanding of the development of motor self-regulation.

## Author Contributions

CK: study design, data collection, data analysis and interpretation, manuscript drafting. IJ: data analysis and interpretation, manuscript drafting. MO: study design, data interpretation, and manuscript drafting. All authors approved the final version to be published.

## Conflict of Interest Statement

The authors declare that the research was conducted in the absence of any commercial or financial relationships that could be construed as a potential conflict of interest.
